# Functional Outcomes Following Posterior Cruciate Ligament and Posterolateral Corner Reconstructions. A Three-year Experience in Seremban, Malaysia

**DOI:** 10.5704/MOJ.2007.017

**Published:** 2020-07

**Authors:** JW Ng, AR Ahmad, GN Solayar

**Affiliations:** 1 Department of Orthopaedics, International Medical University, Kuala Lumpur, Malaysia; 2 Department of Orthopaedics, Hospital Tuanku Ja'afar, Seremban, Malaysia; 3 Department of Orthopaedics, International Medical University, Seremban, Malaysia

**Keywords:** posterior cruciate ligament (PCL) injury, posterolateral corner (PLC) injury, PCL reconstruction, modified larson’s reconstruction, functional outcomes

## Abstract

**Introduction:**

This study was conducted to evaluate the demographics, causes and outcomes of patients who underwent Posterior Cruciate Ligament (PCL) reconstruction and/or Posterolateral Corner (PLC) reconstruction performed at our institution over the last three years. Sub-analysis was performed to assess the impact of delay from injury to surgery and how this affected outcomes.

**Material and Methods:**

From an initial number of 10 patients, seven were contactable and available for analysis. All patients underwent PCL and/or PLC reconstruction (modified Larson’s procedure) between 2017 and 2019. The mean age of our cohort was 31.4±9.6 years (range, 21 to 46). Assessment of functional outcomes pre- and post-operatively were done using the Lysholm knee scoring scale, the Knee injury and Osteoarthritis Outcome Score (KOOS) and visual analogue scale (VAS). The mean follow-up from operation at time of reporting was seven months (range, 2 to 12 months).

**Results:**

There were four combined PCL and PLCs, two isolated PLCs and one patient who underwent an isolated PCL reconstruction. There were significant improvements between pre-operative and post-operative in all functional outcome scores utilised following PCL reconstruction and/or modified Larson’s reconstruction. Lysholm knee scoring scale improved from pre-operative to post-operative at 41.14±12.32 to 74.86±13.52 (p=0.0001), KOOS from 49.71±11.19 to 71.43±13.84 (p=0.001), and VAS from 5.71±2.06 to 2.86±2.48 (p=0.001). Our sub-analysis showed that higher functional outcomes were present when surgery was done less than six months from the time of index injury. There were no complications (eg. Infections, revisions) in this cohort at the time of reporting.

**Conclusion:**

Reconstructive surgery for PCL and/or PLC injury is successful in increasing the functional outcomes of patients post-operatively. Delays from injury to surgery remains a problem in the public setting as patients may need to await appropriate imaging and approval of funding. Increased awareness for early surgical intervention may improve overall outcomes of PCL and/or PLC reconstruction in Malaysia.

## Introduction

The posterior cruciate ligament (PCL) is one of the strongest ligaments found in the knee, and it acts as a primary stabiliser for the posterior part of the joint^1^. It works closely with the structures of the posterolateral corner (PLC) to prevent posterior translation of the tibia and rotational instability. Structures that constitute the PLC are the fibular collateral ligament, popliteofibular ligament, popliteus tendon, arcuate ligament and extracapsular iliotibial band. Injuries of the PCL are normally associated with a concomitant PLC injury however, these are uncommon when compared to anterior cruciate ligament injuries^2^. If these (PCL and PLC) injuries are not managed appropriately, they may lead to chronic knee instability and functional disability. Partial PCL tears associated with a low grade PLC injury may be managed by non-operative treatment such as physical therapy with acceptable results in most cases. On the other hand, reconstructive surgery would be indicated when there is a complete PCL tear and a high grade PLC injury^1^.

This study retrospectively evaluated the outcomes of patients who underwent arthroscopic PCL reconstruction and/or modified Larson’s PLC reconstruction at our institution being the state referral centre for Negeri Sembilan. We hypothesised that shorter time between injury to surgery would result in improved early functional outcomes compared to delayed reconstruction.

## Materials and Methods

A total of 10 patients with isolated PCL tear, isolated PLC or combined injury who underwent PCL reconstruction and/or modified Larson’s reconstruction between 2017 and 2019 in Hospital Tuanku Ja’afar, in Seremban were identified. Of these, seven patients were retrospectively analysed (the rest were uncontactable). Inclusion criteria were patients aged more than 18 years old, presented with knee pain or instability, examination findings of positive posterior drawer test, positive posterior sag, positive varus stress test and magnetic resonance imaging (MRI) findings consistent with PCL tear. Exclusion criteria were revision or bilateral PCL reconstruction, combined ACL and PCL tear, PCL avulsion fracture, medial collateral ligament injury and knee osteoarthritis prior to surgery. Acute ligament injury was defined as a tear that occurred within three weeks prior to surgery; the rest were deemed as chronic injuries. Prior to surgery, options of non-operative and operative managements were discussed with all patients. All surgical procedures were carried out by a single, fellowship trained surgeon (G.N.S.).

The patient was placed in a supine position and under general anaesthesia. Prior to surgery, the affected knee was examined to assess degree of posterior and rotational instability. High tourniquet was used during the procedure. Knee arthroscopy was performed to identify the PCL disruption. PCL reconstruction was performed first in cases where a combined procedure was performed. All PCL reconstruction cases were performed using allograft (Achilles tendon) via an all arthroscopic single bundle reconstruction technique (reconstructing the anterolateral bundle). Following, PCL reconstruction, reconstruction of the PLC using modified Larson’s technique was initiated. The graft used for PLC reconstruction was either allograft (if combined procedure was performed) or by harvesting patient’s ipsilateral semitendinosus tendon as autologous graft (if PLC performed as sole procedure)^3, 4^.

Post-operatively, all patients were kept in a brace locked in extension and given modalities to reduce swelling. They were regularly followed up by both orthopaedic surgeon and sports physicians at the institution and a PCL specific brace was employed by week 2 post-operation.

Outcome evaluations were done through functional assessment. The functional assessment consisted of the Lysholm knee scoring scale, visual analogue scale (VAS) and the Knee injury and Osteoarthritis Outcome Score (KOOS) with subscales of Pain, Symptoms, Activities of Daily Living (ADL) and Quality of Life (QOL). All the assessments were done retrospectively for pre-operative and at post-operative scores in all patients.

The statistical analysis was performed by using SPSS software [ver. 25.0; Chicago, Illinois, USA]. Comparisons in between pre-operative and post-operative functional assessment scores were performed using the paired Student *t* test. Continuous variables were reported as mean values followed by standard deviation. P values of less than 0.05 were considered to be statistically significant.

## Results

The mean age of the seven patients was 31.4±9.6 years, ranging from 21 to 46 years. Four patients were in between 21 to 30 years old, one was in between 31 to 40 years old and the remaining two were above 40 years old. Six of the patients (86%) were male and all patients were Malay. Motor-vehicle accidents were the most common injury mechanisms which accounted for six patients while one patient suffered from workplace injury.

Four patients had combined PCL and PLC injuries, two had isolated PLC injuries and the remaining one patient had an isolated PCL tear. Four patients sustained injuries on the left knee and the other three were on the right knee. The mean duration from injury to surgery was 10.4±4.4 months, ranging from 5 to 17 months. Only one patient had reconstruction surgery done in within six months of injury. No surgical complications (eg. Infection, revision, failure of graft) were reported in this cohort.

Pre-operatively, the mean Lysholm knee scoring scale was 41.14±12.32. The lowest Lysholm knee scoring scale was 21% and the highest was 57% pre-operatively. There were significant improvements post-operatively with a mean score of 74.86±13.52 (p=0.0001). Post-operatively, the highest score was 92% and the lowest was 57%. KOOS also increased significantly from 49.71±11.19 pre-operative to 71.43±13.84 post-operatively (p=0.001). KOOS ranged from 32% - 63% at pre-operative to 54% - 92% at post-operative. The mean VAS pre-operatively was 5.71±2.06 (range, 3 to 8). This improved significantly to a mean score of 2.86±2.48 (range, 0 to 6) post-operative (p=0.001) (Table I).

**Table I:** Summary of patients’ demographic characteristics cause of injury, delay to surgery and functional outcomes

Patient	Operation	Mechanism of Injury	Delay to Surgery	Lysholm		KOOS		VAS	
				Pre	Post	Pre	Post	Pre	Post
1	PCL	MVA	17 months	40	75	57	81	3	0
2	PLC	MVA	7 months	21	58	32	57	8	4
3	PLC	MVA	8 months	57	92	63	92	5	0
4	PCL+PLC	MVA	9 months	44	74	46	68	3	1
5	PCL+PLC	MVA	5 months	42	88	47	81	7	5
6	PCL+PLC	Occupational accident	10 months	31	57	42	54	7	6
7	PCL+PLC	MVA	17 months	53	80	61	67	7	4

## Discussion

Sixty percent of PCL injuries are complicated by a concomitant PLC injury^5^. Persistent knee instability and chronic pain are the main reasons for the patient to undergo reconstructive surgery. Our study aims to evaluate the outcomes of patients undergoing arthroscopic single bundle posterior cruciate ligament reconstruction and/or posterolateral corner reconstruction and to determine whether time interval between injury and surgery affects the results at this single institution.

Motor vehicle accidents and sports are two well-known causes of PCL injury. In Malaysia, Che Ahmad *et al*^6^ revealed that 67% of the patients with PCL tear sustained motor vehicle accidents. According to Caldas *et al*^7^ and Schulz *et al*^8^ motor vehicle accidents were the leading factor for isolated and combined PCL injuries which accounted for 78.8% and 45% of their cohort. On the other hand, Bernhardson *et al*^9^ suggested that 78% of the patients suffered the PCL injury as a results of trauma due to sports event. In our study, motor vehicle accident was the main cause of injuries in the majority of our patients (6 out 7).

Many studies have shown that PCL reconstruction with or without a modified Larson’s gives rise to significant improvements in terms of functional outcomes^2, 10-12^. A systematic review from 2000 to 2016 by Petrillo *et al*^5^ revealed that PCL and PLC reconstruction had led to an increase in the Lysholm score from 54.7 ± 9.1 to 83.2 ± 4.9 in 66 patients. Kim *et al*^13^ evaluated outcomes of combined PCL and PLC reconstructions in 42 patients and showed better results in Lysholm functional scores during final follow-up at two to six years when compared to pre-operation. Another study done by Kim *et al*^14^ showed Lysholm scores also rose remarkably from pre-operative 59.63 ± 4.49 to post-operative 83.04 ± 5.68 in combined reconstruction. Mygind-Klavsen *et al*^2^ and Lind *et al*^15^ also reported that there were significant improvements in KOOS from pre-operative to final follow-up. From our study, both Lysholm knee scoring scale and KOOS showed significant improved differences pre- and post-operatively; with similar results as shown by other authors internationally.

The optimal time from injury to surgery remains one of the controversial issues in the outcomes of patients as mentioned earlier^16-18^. Early interventions may allow earlier rehabilitation and mobility of the knee joint and thus, has the potential of improving overall success rates following reconstruction. However, higher risks of arthrofibrosis has been reported in early reconstruction cases and this may be a reason to opt for delayed reconstruction^16^. Hohmann *et al*^17^ have reported that early surgical reconstruction for injuries of knee ligaments had markedly better outcomes in term of Lysholm scores than late surgical intervention. Conversely, Rusdi *et al*^18^ reviewed that there was no association between the optimal time of surgery and patients’ Lysholm scores. Our study suggested that higher functional outcomes were present when the reconstructive surgery was done less than six months from injury. A longer duration of more than six months from injury to surgery in most of the patients were due to delays in obtaining appropriate imaging and funding approval for surgical intervention.

This study has several limitations. The small number of patients makes it difficult to make strong conclusions on the effect of delayed surgery however, our results do favour earlier intervention. We acknowledge that heterogenicity of aetiologies (PCL, PLC and combined) may foreshadow direct comparison however, as there is a paucity in the Malaysian body of literature at present with regards to outcomes of these injuries, we opted to include them in this review.

## Conclusion

Reconstructive surgery for PCL and/or PLC injury is successful in increasing the functional outcomes of patients post-operatively. Delays from injury to surgery remains a problem in the public setting as patients may need to await appropriate imaging and approval of funding. Increased awareness for early surgical intervention may improve overall outcomes of PCL and/or PLC reconstruction in Malaysia.
